# Taking aim at refractive error

**Published:** 2024-05-15

**Authors:** Amanda Davis, Scott Mundle

**Affiliations:** 1 IAPB Refractive Error Work Group Co-Chair, Sydney, Australia.; 2 IAPB Refractive Error Work Group Co-Chair, Winnipeg, Canada.

**Refractive errors remain the leading cause of visual impairment globally. What is needed to ensure access to spectacle correction for everyone?**

**Figure:**
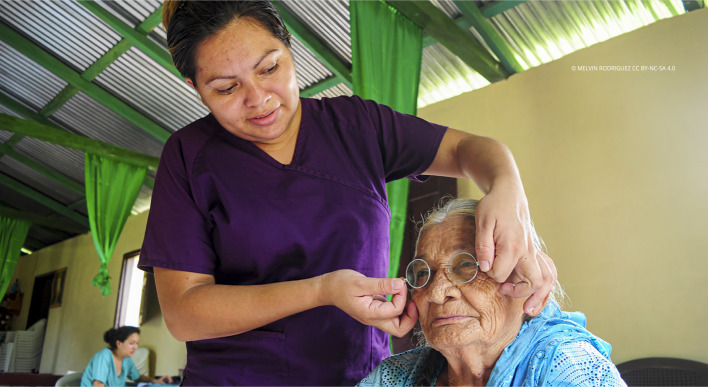
Good vision in older people has a significantly positive impact on their physical and mental health and their functional ability. Nicaragua

Globally, uncorrected refractive errors are the main cause of visual impairment.^1^ This translates to millions of people being negatively impacted in their lives due to lack of access to services and spectacles, resulting in lost education and employment opportunities, lower productivity, and impaired quality of life.

There is an urgent need for community-based strategies to tackle the rising prevalence of refractive errors and ensure equitable access to eye care services for all individuals, regardless of age, gender, socioeconomic status, or geographical location. By the year 2050, it is estimated that half the global population will be affected by myopia; this, as well as the emergence of myopic macular degeneration as an increasing cause of vision impairment, will require a significant focus on preventative and treatment strategies. In addition, with the global population growing and people living longer, there will be a significant increase in the number of people who need near vision spectacles for presbyopia.

The *Community Eye Health Journal* has produced this issue on refractive errors to further build on the knowledge of the eye care community, to address the pressing needs of the estimated 1 billion people worldwide who have vision impairment because they do not have access to a pair of spectacles. Rapid action is needed, which is why – in 2021 – the World Health Organisation member states endorsed a global target to increase effective refractive error coverage (eREC) by 40 percentage points.

In addition to articles about global magnitude of refractive error and the WHO SPECS 2030 initiative to help countries develop a solution and meet the 40 percentage point target, readers can also find specific guidance on managing various refractive conditions, including hyperopia, myopia, and presbyopia. Each condition presents unique challenges and requires tailored interventions to optimise visual outcomes and enhance quality of life for those affected.

Through evidence-based recommendations and practical insights, these articles aim to equip eye health practitioners, managers, and policy makers with the knowledge and skills needed to support the management of refractive errors in their respective settings. This issue also provides valuable guidance on practical skills essential for delivering comprehensive refractive error care in the community. From guidance on performing cycloplegic refraction in school children to ensuring the correct prescription and fitting of spectacles, these skills are indispensable for promoting and supporting quality eye health outcomes.

## School eye health

School health programmes are a vital opportunity to deliver eye health promotion, screening, examination, provision of glasses, and other services. To support the implementation of vision screening for children, the WHO released the Vision and eye screening implementation handbook (bit.ly/WHOscreen). For specific guidance on the implementation of a school eye health programme, the IAPB is revising and updating its current guidelines. These new guidelines will be launched at the IAPB 2030 Insight Live event in June 2024; the current guidelines are available at bit.ly/IAPBschool

## A vision for the future

We need to work together to ensure that no individual is left behind in their journey towards optimal vision and well-being. By advocating for policies that promote equitable access to eye care services, investing in training and capacity building initiatives, and fostering partnerships with local stakeholders, we can create sustainable solutions that address the systemic barriers to accessible and quality refractive error services and improve eye health outcomes. Through collaboration, innovation, and a shared commitment to excellence and integration, we can build more robust systems and healthier, more resilient communities for the future.
